# Retraction: Broadly neutralizing monoclonal antibodies against influenza A viruses: current insights and future directions

**DOI:** 10.3389/fmicb.2026.2026341

**Published:** 2026-10-20

**Authors:** Frontiers Editorial Office

The journal retracts the article cited above.

Following publication, concerns were raised regarding the scientific validity of the article. An investigation was conducted in accordance with Frontiers' policies.

It was found that the complaints were valid and that the article does not meet the standards of editorial and scientific soundness for Frontiers Microbiology - Virology; therefore, the article has been retracted.

This retraction was approved by the Chief Editors of Frontiers in Microbiology and the Chief Executive Editor of Frontiers. The authors do not agree to this retraction.

Frontiers would like to thank the concerned reader who contacted us regarding the published article.

